# Supplementary material for: Successful outcomes with low–threshold intervention for cannabis use disorders in Norway - an observational study
Source: PLoS One. 2022 Jun 16;17(6):e0269988. doi: 10.1371/journal.pone.0269988 (PMC9202853; doi:10.1371/journal.pone.0269988)
Supplement: S2 File — (PDF) [file pone.0269988.s002.pdf]

Hap-2: A national multicenter project to investigate the short- and long-term effects of a standardized intervention for cannabis smoking cessation

1. Introduction

Cannabis use is a hotly discussed topic. The merits of medical marijuana and the decriminalization and legalization of recreational marijuana use are politically and scientifically debated, but cannabis remains the most widely used illicit drug worldwide. Globally in 2013, 181.8 million people aged 15–64 years used cannabis for nonmedical purposes; in many developed countries, people first use marijuana in their mid-teens [1]. Cannabis-use disorders are defined similar to other potentially addictive agents (e.g., opioids and amphetamines) and comprise a cluster of behavioral, cognitive, and physiological phenomena that develop after repeated cannabis use [2]. There are some indications that the prevalence of cannabis dependence increased worldwide between 2001 and 2010, with an estimated 13.1 million people now dependent on cannabis [3].

In Norway, figures from the National Criminal Investigation Service (KRIPOS) in 2015 revealed that the amount of confiscated cannabis products was unchanged relative to previous years [4]. Tetrahydrocannabinol (THC, the primary psychoactive compound in cannabis) was detected in 35% of drivers suspected of driving under the influence of cannabis; thus, cannabis is one of the most frequently detected illicit drugs in drivers in Norway [5]. According to the survey “Young in Oslo” the proportion of 15- and 16-year-old adolescents who have ever tried cannabis fell from 13% in 1996 to 9% in 2006 and 2012 [6]. Twenty-nine percent of respondents 16–34 years old had used cannabis at least once in their lifetime. Among respondents 16–24 years old, 12% had used it in the last 12 months and 5% within the previous 30 days.

Withdrawal syndrome has been well documented in people with cannabis dependence [7]. Regular cannabis users can develop dependence on the drug: the risk may be around one in ten among those who use cannabis at all, one in six among adolescent users, and one in three among daily users [7-9]. There is growing evidence that regular, heavy cannabis use during adolescence is associated with more severe and persistent negative outcomes than use during adulthood. Daily use by adolescents and young adults is associated with a variety of negative health and psychological outcomes such as increased risk of anxiety and depressive symptoms and increased rates of suicidal ideation and behavior. In addition, there may be a dose-response relationship between cannabis use during adolescence and the risk of developing psychotic symptoms or schizophrenia [8, 9].

A variety of educational and social risk factors are associated with regular cannabis use, including dropping out of school. The most significant consequence of regular cannabis use may be the negative impact on education and learning due to reduced cognitive capacity, lack of motivation, and increased risk of using other illicit drugs [10-12]. While almost all adolescents enroll in upper secondary education in Norway, only two-thirds complete this educational level within a time period of 5 years. Adolescents drop out of school for many reasons, and the impact of cannabis use on dropping out is probably underestimated [11]. Cannabis affects young brains during the most sensitive period of brain development and maturation; it reduces cognitive function, prevents learning, distorts memory, and affects the ability to make reasoned decisions [13-15]. The high dropout rate is associated with considerable expense for both the dropouts and society in terms of loss of income, weaker attachment to the labor market, and greater utilization of various social security and welfare

resources. A cost–benefit analysis from 2010 showed that the net social costs of dropouts from the educational system in Norway equals approximately 900,000 NOK (110,000 €) over the individual's lifetime [16]. This analysis took into account both private earnings and public expenditures related to the educational level attained by recipients. For example, the proportion of welfare benefit recipients among dropouts is significantly higher than among the general population. The findings described above imply that the total net social gain achieved by reducing the number of dropouts by one-third compared to the current level is approximately 5.4 billion NOK (750 million €) per yearly age cohort. The income loss of individuals is the largest cost component of dropouts.

2. Description of needs

Although survey data have shown a downward trend in cannabis use, demands for help to address regular cannabis use and cannabis dependence are rising in Europe [17]. This increased need must be considered within the context of how services are provided and how referrals are made to programs and specialists, but there is some speculation as to whether the increase in users who are seeking treatment may be linked to the increasingly higher amounts of THC in cannabis products. There has been an upward trend in the mean THC content of confiscated cannabis preparations in the USA and some European countries [7, 8, 18]. Meanwhile, the number of cannabis users seeking help from outpatient services at the Addiction Unit (ARA) of Sørlandet Hospital rose 50% from 2011 (n = 231) to 2015 (n = 348) [19]. Thus, there is increasing demand for treatment of this condition. In general, outcome studies of interventions for cannabis use have only investigated treatment methods that were developed for other addictive drugs. These methods include cognitive behavioral treatment, motivational interviewing, and contingency management [20]. Although a number of pharmaceuticals reduce the cravings and/or relapse associated with addictive drugs, none have been approved for use in people with cannabis dependence or withdrawal [7, 8].

Psychosocial approaches to the cessation of cannabis use appear to have only moderate short-term benefits and significant rates of relapse. The results for abstinence as a long-term outcome are disappointing; the quit rate is rarely above 20–25% at 12 months follow-up, similar to that seen for tobacco smokers [20]; most cannabis smokers also smoke tobacco [21]. The equivalent finding among tobacco smokers who try to quit on their own is that about half start smoking again within a week and only one in 20 is nicotine free after 1 year [22]. Quit rates subsequent to professional interventions ought to at least exceed the quit rates for those who try to quit on their own. However, even when the best pharmacological and psychosocial means are available, quit rates seldom exceed 25% [23]. Programs associated with quit rates that exceed these levels should be considered promising for both cannabis and tobacco use. The World Health Organization has requested that better and more tailored interventions be developed specifically to treat cannabis-use disorders [7]. Group-based therapy is generally thought to be more efficient than individual therapy [24], but because of the particular cognitive distortions associated with cannabis use, there is a debate as to whether individual therapy for chronic cannabis use may be superior [20, 25].

The HAP model

In 1995, Lundqvist described disruptions in cognitive processes after chronic use of cannabis preparations. His findings provided support for the idea that there are specific patterns of thinking that can be considered cannabis state–dependent sets of cognitive processes [26, 27]. These patterns can be considered a component of a mental and behavioral profile unique to cannabis use compared to use of heroin or amphetamines. Cannabis induces loss of internal

control and cognitive impairment, especially of attention and memory, for the duration of intoxication. Heavy cannabis use is associated with reduced function of the attentional/executive system, exhibited by decreased mental flexibility, increased perseveration, reduced learning, and reduced ability to shift and/or sustain attention [26]. Lundqvist also explored how the effects of cannabis on the human brain can be optimally addressed by therapists. His objective was to draw upon knowledge of neuropsychology, cognitive psychology, and social psychology combined with clinical observations to understand the fragmentation of the neuropsychological network caused by cannabinoids and their effects on the daily lives of individuals [26].

Lundqvist and Ericsson developed a treatment model based on Lundqvist's findings. The model is a manual-based outpatient program specially tailored to cannabis-dependent smokers and the unique patterns of thought associated with chronic cannabis use [28]. The program is called the Cannabis Cessation Program (Hasj Avvennings Programmet, or HAP), and it is primarily based on one-to-one consultations (individual based, or I-HAP). The method uses a combination of cognitive therapy and psychoeducation. The program period covers the normal withdrawal period for cannabis smoking cessation (8 weeks) and comprises 15 meetings. There are three phases: medical, psychological, and social. During each of these phases, the participants focus upon different themes. The program is engineered to help participants identify and handle the physical and emotional changes that take place within themselves during the various phases, and help them handle the social challenges that arise. The overarching aim of the program is to help cannabis smokers reorganize the thinking pattern that developed while they were using cannabis and to increase their social and psychological coping skills. The program focuses in particular on how cannabis affects the ability to solve everyday problems. The manual has been translated into eight languages. It has been used in Sweden since 1995 and in Norway since 2005 [29]; however, the results of this method have not been thoroughly evaluated.

To implement the HAP program in Norway, the Addiction Unit of Sørlandet Hospital, in cooperation with the Health and Social Services of Kristiansand municipality, established a center for cannabis treatment expertise and cannabis cessation programs ("Cannabisforum Sør," established in 2005). The aims of the center were to ensure that staff could competently administer the HAP model and to train and offer clinical guidance to the surrounding municipalities.

In cooperation with the University of Agder (UiA), the Addiction Unit of Sørlandet Hospital and the Health and Social Services of Kristiansand municipality have established a half-year course pertaining to cannabis abuse, prevention, and treatment. The course also focuses specifically on the HAP model. To date, 330 professionals from all over Norway have completed the course. The center in Kristiansand has become a leader in Norway with respect to clinical work and research on the HAP model. Its ideas and knowledge are communicated to other parts of Norway via an established national HAP network. Local networks have also emerged in locations such as Oslo and Nord-Trøndelag County. Moreover, local workshops have been held all over the country and are led by lecturers from the Kristiansand center. Eight educational films have been made to train professionals and for use in the HAP. There is a yearly congress, "Out of the Fog" (Ut av Tåka Seminaret), which has taken place in Kristiansand since 2005, with 300–400 participants each year, to support more widespread use of the model. In addition, there is a website that explains the methodology and is used to establish contact with new centers: www.hasjavvenning.no.

In short, the actual clinical work occurs mainly at the community level (i.e., in municipalities), while the involved specialized health services focus on competence building, clinical supervision, support, and research. This type of cooperation between municipalities and specialist services meets the requirements outlined by the Norwegian health authorities in the Coordination Reform guidelines ("Samhandlingsreformen") with respect to increasing competence and cooperation among different levels of services [30, 31].

The results of the study described herein will have implications for clinical practice and could be used to adapt the HAP model to outpatient addiction services and accelerate its adoption by municipalities that could benefit from it.

3. Hypotheses, aims, and objectives

The study will examine short- and long-term outcomes following treatment with the HAP model: immediately after the program, after 3 months, and after 12 months. The **primary outcome** will be changes in cannabis use and abstinence in the 30 days prior to the assessments. Based on a previous HAP pilot study [32], a central question is whether positive results observed in the short term persist at 12 months follow-up. **Secondary outcomes** of the study include changes in the use of other drugs and alcohol, changes in mental health, quality of life and sense of coherence, school attendance, changes in the number of days spent working, use of social welfare benefits, and reductions in criminal behavior. Related questions include the following: Do cannabis-dependent smokers quit cannabis smoking but switch to alcohol or other drug use? What is the prevalence of synthetic marijuana use among cannabis smokers, and do those who use synthetic marijuana have a worse prognosis? Will cannabis users who have dropped out of school re-enter the educational system?

4. Project methodology

4.1 Project arrangements, selection of methods, and analyses

Inclusion criteria

- Chronic cannabis users with a score > 4 on the Severity Dependence Scale (SDS, see below).
- Age > 16 years. Based on the intake criteria of the treatment centers, the main target group will be between 18 and 30 years. However, some centers also include participants > 30 years old.
- Both men and women will be included.

Exclusion criteria

- Polydrug use in which use of drugs other than cannabis predominates.
- Severe psychiatric or physical disorders.
- Severe levels of criminal behavior.

Experimental design, participants, and researchers

The study has as prospective, observational design. All centers that use HAP have been invited to participate in data collection. The participating centers have been trained in the scoring of forms, interpretation, and data transfer. Participants are interviewed and surveyed before they begin the program to ensure that they meet the inclusion criteria. After providing written informed consent, participants complete the inventory described below. Participants will be reassessed with the same survey after they have completed the program and at 3 and

12 months follow-up. Efforts will also be made to examine non-completers after 3 and 12 months.

The key coordinator at each location transfers the data into a web-based survey, which will be stored in a common database through a secure sockets layer (SSL). Individuals are not directly identifiable; a code for each individual is used and no direct person-identifiable data transmitted. Each course location stores a manual code list in locked cabinets, and the identity of the participants is not made known to the project team. Data collection began in 2014 and to date we have included 109 participants from the municipalities of Kristiansand, Oslo, and Fredrikstad. The PhD fellow will continue the data collection and encourage other centers to participate.

Instruments
In addition to basic demographics, the inventory includes the items and scales below:

Primary outcome

- Changes in cannabis use and abstinence in the 30 days prior to assessment, based on measurements of cannabis use in the previous 30 days as measured by a question from the European version of the Addiction Severity Index (EuropASI) [33].

Secondary outcomes

- Mental distress, measured with the Symptom Check List-25 (SCL-25), which comprises 25 questions with a four-point response scale that maps anxiety and depression and yields a global score for mental distress [34, 35].
- Perceived substance use of cannabis, nicotine, alcohol, and other addictive substances, measured with visual analog scales [36, 37].
- General well-being, measured with the Outcome Rating Scale (ORS) [38].
- Number of working days.

In addition, we also use the following scales to provide information about inclusion criteria and/or possible predictors of outcome:

- Severity of cannabis dependence measured with the SDS, which contains five items scored on a four-point Likert response scale [39-41]. The scale is used to assess the eligibility of respondents and to control for severity in the analyses due to the quasi-experimental design (see Statistics section).
- Sense of coherence, measured with the Sense of Coherence scale (SOC) [42, 43].

Statistics
Baseline variables will be reported with descriptive statistics. Paired sample t-test will be used to examine changes in continuous variables between the time points and the McNemar test will be used to examine changes in binomial variables. The results for secondary outcomes will be assessed with similar statistical tests.

4.2 Participants, organizations, and collaborators
Project group:

- Principle investigator and PhD supervisor: John-Kåre Vederhus PhD, Addiction Unit (ARA), Sørlandet Hospital HF (SSHF)
- Senior psychiatrist/senior research fellow: Øistein Kristensen, ARA, SSHF
- Leader of HAP Kristiansand: Madelene Skårdal MSc, Kristiansand municipality

- Coordinator of HAP, Oslo: Malin Rørendal MSc, Rusmiddeletaten Oslo municipality
- Leader of Cannabisprosjektet in Fredrikstad: Kine Norbom, Fredrikstad municipality
- Assistant supervisor: Professor Thomas Clausen MD/PhD: ARA SSHF and University of Oslo
- International supervisor: Professor Thomas Lundqvist PhD: Lund University Hospital, Sweden
- User representative: Jan Ivar Ekberg, "A-larm", a national addiction related user-organization
- Statistical supervisor: Are Hugo Pripp PhD: Oslo Centre of Biostatistics and Epidemiology, Oslo University Hospital

Preconditions for research

The Clinic for Psychiatry and Addiction Medicine (KPA), Sørlandet Hospital, has a large research portfolio. There are currently ten ongoing PhD research fellowships, and two associate professor positions have been established. Statistician Supervisor Are Hugo Pripp, Oslo University Hospital/Sørlandet Hospital, has supervised previous projects at the site of the current study.

The present clinical study is being conducted by the Research Department at ARA, Sørlandet Hospital. ARA is a subunit of KPA and has been carrying out addiction research since 1987. In the beginning, this was mainly naturalistic outcome research. Later, intervention studies, randomized controlled trials, and registry studies also took place [46-48]. A research unit was established in 2001 and currently comprises a research leader (MD/PhD), a research fellow (PhD), a research assistant (MPH), and a clinical nurse specialist (CNS, MPH). Thomas Clausen MD, PhD from the Norwegian Center for Addiction Research (University of Oslo, UiO) acts as a research supervisor. Four PhDs at ARA have graduated via UiO or the University of Bergen (UiB). The studies of two of these students were supported by grants from the Norwegian Research Council [49, 50]. At present, there are two ongoing PhD fellowship studies at ARA: a follow-up study of patients with substance-use disorders patients who were involuntarily committed to treatment, and a study of relatives of SUD patients. An extensive list of ARA publications is available at http://avhengighetsbehandling.no/html/publ-hoved.html.

ARA is taking part in some other ongoing cooperative projects. One of the projects is another cannabis project that is being run at ARA, called "Method trials for detection of synthetic cannabinoids in blood and urine." This study is a partnership between ARA and the Department of Pharmaceuticals, St. Olavs Hospital, Trondheim/NTNU, supervised by Professor Olav Spigset. In addition to extensive national collaborations, previous collaborations include collaborations with researchers at Stanford University (Professor Keith Humphreys and Associate Professor Christine Timko) [48, 51]. For the present study, we are cooperating with the developer of the HAP model, Thomas Lundqvist, at Lund University Hospital in Sweden and also with the extensive Swedish network that addresses chronic cannabis use. Funding of the HAP-2 PhD fellow will help us to perform follow-up interviews with HAP participants, do analyses, and write up the results of the study under the supervision of qualified academic personnel.

4.3 Budget

PhD fellowship: 3 years, from 2017 to 2019. NOK 1,039,000 × 3 = NOK 3,117,000.
Expenses (e.g., travel expenses, meetings, information technology expenses, and supervision): NOK 100,000 × 3 = 300,000.

4.4 Plan for activities, visibility, and dissemination

We have outlined a milestone plan (activities) for the project in the application form (eSøknad).

- Autumn 2016: Apply for funding. Relevant permissions have already been obtained, and data collection is ongoing.
- Spring 2017: Find qualified PhD candidate as soon as funding is secured. Qualified graduates (with master's degrees) are currently doing clinical work at ARA. They are expected to apply for the position and will be able to start at relatively short notice. The inclusion of participants (expected to be completed by mid-2017) and data collection from follow-up visits (expected to be completed by mid-2018) will continue.
- Autumn 2017 to spring 2019: Write up results and submit papers for publication.
- Spring 2019 to autumn 2019: Write summary of PhD thesis research. Submit PhD thesis.

Descriptions of the support needed to efficiently implement the project (data collection, analyses, interpretation of results, drafting of manuscripts, and preparation for PhD dissertation) appear above ("Preconditions for research"). Additional remarks are as follows: Data collection is ongoing. To date, 60% of the required respondents have been enrolled. Based on the data collection thus far, we expect the inclusion period to last for one more year (until mid-2017); follow-up data collection will be finalized summer 2018. The planned time frame allows for time to write up the results and publish papers beginning early in the PhD period. This is an effective use of time that will allow the PhD student to complete the required work within the allocated timeframe.

4.5 Plan for implementation

At least three scientific papers will be written to form the basis for a PhD dissertation. We intend to publish them in international peer-reviewed journals, such as *Substance Abuse Treatment, Prevention, and Policy*; *Addiction Science & Clinical Practice*; *Journal of Substance Abuse Treatment*; and *Drug & Alcohol Dependence*. The project will also generate papers in addition to those required for the PhD dissertation. The results will be communicated in relevant national and international research arenas and targeted to decision makers in the addiction and health fields, as well as to the existing clinical network and their associated websites and social media sites. Moreover, the results will be used to improve the cannabis abuse, prevention, and treatment course at the UiA and to increase dissemination of the HAP model by providing further documentation of its effects.

Tentative titles for the papers are as follows:

1. "Changes in cannabis use after an 8-week program for cannabis cessation"
2. "Predictors of abstinence from cannabis use after completion of an 8-week cannabis cessation program"
3. "Follow-up results 3 months after an 8-week intervention program for cannabis cessation"

5. User involvement

Former users the HAP program participated in discussions regarding research questions, outcome measures, timeframes of the evaluation (i.e., times at which follow-up interviews are conducted), design of information brochures, and questions about promoting participation in the project and addressing issues that may arise. In addition, user involvement includes formal participation in the project group by a representative of the User Board of the KPA (Jan Ivar Ekberg of "A-larm").

6. Ethics

Participants will be included after they have provided written informed consent to join the study. The study was approved by the Regional Ethics Committee (No. 2012/1407).

7. References

1. UNODC. *World drug report 2015*. Vienna: United Nations Office on Drugs and Crime; 2015.
2. American Psychiatric Association. *Diagnostic and statistical manual of mental disorders : DSM-5*. 5th ed. Washington, D.C.: American Psychiatric Association; 2013.
3. Degenhardt L, Ferrari AJ, Calabria B, Hall WD, Norman RE, McGrath J, *et al.* The global epidemiology and contribution of cannabis use and dependence to the global burden of disease: results from the GBD 2010 study. *PLoS One* 2013,**8**:e76635.
4. KRIPOS. Drugs and steroid statistics 2015 [Narkotika- og dopingsstatistikk 2015]. Oslo: KRIPOS; 2015.
5. Folkehelseinstituttet. Facts about cannabis & marijuana [Fakta om cannabis, hasjisj og marihuana]. Oslo: Folkehelseinstituttet; 2016.
6. Øia T. *Young in Oslo. [Ung i Oslo 2012 : nøkkeltall]*. Oslo: Norsk institutt for forskning om oppvekst; 2012.
7. World Health Organization. The health and social effects of nonmedical cannabis use. Geneva, Switzerland; 2016.
8. Hall W. What has research over the past two decades revealed about the adverse health effects of recreational cannabis use? *Addiction* 2015,**110**:19-35.
9. Volkow ND, Baler RD, Compton WM, Weiss SR. Adverse health effects of marijuana use. *N Engl J Med* 2014,**370**:2219-2227.
10. Silins E, Fergusson DM, Patton GC, Horwood LJ, Olsson CA, Hutchinson DM, *et al.* Adolescent substance use and educational attainment: An integrative data analysis comparing cannabis and alcohol from three Australasian cohorts. *Drug Alcohol Depend* 2015,**156**:90-96.
11. Simonsen B. Hash som pædagogisk problem – i ungdomsuddannelserne. Odense: Erhvervsskolernes Forlag; 2014.
12. Vedøy TF, Skretting A. *Ungdom og rusmidler : resultater fra spørreskjemaundersøkelser 1968-2008*. Oslo: SIRUS; 2009.
13. Meier MH, Caspi A, Ambler A, Harrington H, Houts R, Keefe RS, *et al.* Persistent cannabis users show neuropsychological decline from childhood to midlife. *Proc Natl Acad Sci U S A* 2012,**109**:E2657-2664.
14. Smith MJ, Cobia DJ, Wang L, Alpert KI, Cronenwett WJ, Goldman MB, *et al.* Cannabis-related working memory deficits and associated subcortical morphological differences in healthy individuals and schizophrenia subjects. *Schizophr Bull* 2014,**40**:287-299.
15. Crean RD, Crane NA, Mason BJ. An evidence based review of acute and long-term effects of cannabis use on executive cognitive functions. *J Addict Med* 2011,**5**:1-8.
16. Falch T, Johannesen AB, Strøm B. The social costs of dropouts in upper secondary education in Norway. Trondheim: NTNU; 2010.
17. Schettino J, Leuschner F, Kasten L, Tossmann P, Hoch E, Ferri M, *et al. Treatment of cannabis-related disorders in Europe*. Lisbon: European Monitoring Centre for Drugs and Drug Addiction (EMCDDA); 2015.
18. EMCDDA. *European Drug Report 2015: Trends and Developments*. Lisbon: European Monitoring Centre for Drugs and Drug Addiction (EMCDDA); 2015.
19. Nilsson C. Administrative data. Kristiansand: Addiction Unit, Sørlandet Hospital; 2016.
20. Gates PJ, Sabioni P, Copeland J, Le Foll B, Gowing L. Psychosocial interventions for cannabis use disorder. *Cochrane Database Syst Rev* 2016:CD005336.
21. Klokkervold B, Wivestad A. Drug use among adolescent students [Rusvaner blant ungdom skoleåret 2013 - 2014. En undersøkelse av alle 10. klasser i Kristiansand]. Kristiansand: Municipality of Kristiansand; 2015.

22. Hughes JR, Keely J, Naud S. Shape of the relapse curve and long-term abstinence among untreated smokers. *Addiction* 2004,**99**:29-38.
23. Cahill K, Lindson-Hawley N, Thomas KH, Fanshawe TR, Lancaster T. Nicotine receptor partial agonists for smoking cessation. *Cochrane Database Syst Rev* 2016:CD006103.
24. Weiss RD, Jaffee WB, de Menil VP, Cogley CB. Group therapy for substance use disorders: what do we know? *Harv Rev Psychiatry* 2004,**12**:339-350.
25. Madigan K, Brennan D, Lawlor E, Turner N, Kinsella A, O'Connor JJ, *et al.* A multi-center, randomized controlled trial of a group psychological intervention for psychosis with comorbid cannabis dependence over the early course of illness. *Schizophr Res* 2013,**143**:138-142.
26. Lundqvist T. Cognitive consequences of cannabis use: comparison with abuse of stimulants and heroin with regard to attention, memory and executive functions. *Pharmacol Biochem Behav* 2005,**81**:319-330.
27. Lundqvist T. Cognitive dysfunctions in chronic cannabis users observed during treatment - an integrative approach. Stockholm: University of Lund; 1995.
28. Lundqvist T, Ericsson D. The theoretical background for the cannabis cessation program [Den teoretiska bakgrunden till Haschavvänjningsprogrammet (HAP) - en metod för behandling av cannabismissbruk]. *Socialmedicinsk Tidsskrift* 2007:37-45.
29. Lundqvist T, Ericsson D. A guide for you who want to quit smoking cannabis [En guide for DEG som vil slutte med hasj]. Norwegian version. Skien: Borgestadklinikken; 2005.
30. Hansen BH (Minister of Health and Social Services Departement). A new health sector reform: The Coordination Reform In; 2008.
31. Hansen BH. The Cooperation Reform ["Samhandlingsreformen"]. St.Meld. nr. 47 (2008-2009); Ministry of Health and Social Services, ed. Oslo; 2009.
32. Hansen AR, Ropstad G, Kristensen Ø, Clausen T. Cannabis smoking cessation courses; feasibility and follow-up results [Kurs i røykeavvenning for cannabisbrukere – en oppfølgingsstudie]. *Sykepleien Forskning* 2011,**6**:368-374.
33. McLellan AT, Cacciola JC, Alterman AI, Rikoon SH, Carise C. The Addiction Severity Index at 25: Origins, Contributions and Transitions. *The American Journal on Addictions* 2006,**15**:113-124.
34. Derogatis LR. *SCL 90 R administration, scoring and procedures manual II for the revised version and other instruments of the psychopathology rating scale series*. Towson, MD: Clinic Psychometric Research; 1986.
35. Sandanger I, Moum T, Ingebrigtsen G, Dalgard OS, Sorensen T, Bruusgaard D. Concordance between symptom screening and diagnostic procedure: the Hopkins Symptom Checklist-25 and the Composite International Diagnostic Interview I. *Soc Psychiatry Psychiatr Epidemiol* 1998,**33**:345-354.
36. Parkin D, Devlin N. Is there a case for using visual analogue scale valuations in cost-utility analysis? *Health Econ* 2006,**15**:653-664.
37. Torrance GW, Feeny D, Furlong W. Visual analog scales: do they have a role in the measurement of preferences for health states? *Med Decis Making* 2001,**21**:329-334.
38. Miller SD, Duncan BL. The outcome rating scale: A preliminary study of the reliability, validity, and feasibility of a brief visual analog measure. *Journal of Brief Therapy* 2003,**2**:91-100.
39. Allsop DJ, Copeland J, Norberg MM, Fu S, Molnar A, Lewis J, *et al.* Quantifying the clinical significance of cannabis withdrawal. *PLoS One* 2012,**7**:e44864.
40. Gossop M, Best D, Marsden J, Strang J. Test-retest reliability of the Severity of Dependence Scale. *Addiction* 1997,**92**:353.
41. Martin G, Copeland J, Gates P, Gilmour S. The Severity of Dependence Scale (SDS) in an adolescent population of cannabis users: reliability, validity and diagnostic cut-off. *Drug Alcohol Depend* 2006,**83**:90-93.
42. Antonovsky A. The structure and properties of the sense of coherence scale. *Soc Sci Med* 1993,**36**:725-733.
43. Eriksson M, Lindström B. Validity of Antonovsky's sense of coherence scale: a systematic review. *J Epidemiol Community Health* 2005,**59**:460-466.

44. Petrell B, Blomqvist J, Lundqvist T. Out of the fog [Ut ur dimman]. Stockholm: Maria Ungdom; 2005.
45. Kim J, Seo BS. How to Calculate Sample Size and Why. *Clinics in Orthopedic Surgery* 2013,**5**:235-242.
46. Kunoe N, Lobmaier P, Vederhus JK, Hjerkinn B, Hegstad S, Gossop M, *et al.* Naltrexone implants after in-patient treatment for opioid dependence: randomised controlled trial. *Br J Psychiatry* 2009,**194**:541-546.
47. Opsal A, Clausen T, Kristensen O, Elvik I, Joa I, Larsen TK. Involuntary hospitalization of first-episode psychosis with substance abuse during a 2-year follow-up. *Acta Psychiatr Scand* 2011,**124**:198-204.
48. Vederhus JK, Timko C, Kristensen O, Hjemdahl B, Clausen T. Motivational intervention to enhance post-detoxification 12-Step group affiliation: a randomized controlled trial. *Addiction* 2014,**109**:766-773.
49. Vederhus JK. Addiction professionals' and substance abuse patients' attitudes towards and usage of 12-step-based self-help groups. Oslo: University of Oslo; 2012.
50. Opsal A. Involuntarily admitted patients with substance use disorders. Oslo: University of Oslo; 2013.
51. Vederhus JK, Clausen T, Humphreys K. Assessing understandings of substance use disorders among Norwegian treatment professionals, patients and the general public. *BMC Health Serv Res* 2016,**16**:52.
