# Supplementary material for: Successful outcomes with low–threshold intervention for cannabis use disorders in Norway - an observational study
Source: PLoS One. 2022 Jun 16;17(6):e0269988. doi: 10.1371/journal.pone.0269988 (PMC9202853; doi:10.1371/journal.pone.0269988)
Supplement: Attachment — Submitted filename: Response to Reviewers_220516.docx [file pone.0269988.s003.docx]

**Responses to the reviewers**

PONE-D-21-34465: Successful outcomes with low–threshold intervention for cannabis use disorders in Norway - an observational study

PLOS ONE

**Editorial comments:**

- This rebuttal letter responds to each point raised by the academic editor and reviewer(s). The letter is uploaded as a separate file labeled 'Response to Reviewers'.
- A marked-up copy of the manuscript has been provided that highlights changes made to the original version. The separate file is labeled 'Revised Manuscript with Track Changes'.
- An unmarked version of the revised paper without tracked changes is also uploaded, labeled 'Manuscript'.

If applicable, we recommend that you deposit your laboratory protocols in protocols.io to enhance the reproducibility of your results. Protocols.io assigns your protocol its own identifier (DOI) so that it can be cited independently in the future. For instructions see: <https://journals.plos.org/plosone/s/submission-guidelines#loc-laboratory-protocols>.

Response: We have submitted the protocol as Supporting Information.

**Journal Requirements:**

1. When submitting your revision, we need you to address these additional requirements.

Please ensure that your manuscript meets PLOS ONE's style requirements, including those for file naming. The PLOS ONE style templates can be found at 
<https://journals.plos.org/plosone/s/file?id=wjVg/PLOSOne_formatting_sample_main_body.pdf>

1. Response: We have adjusted the manuscript according to the PLOS ONE’s style template. The tables are placed within the text, as specified.

2. We note that you have indicated that data from this study are available upon request. PLOS only allows data to be available upon request if there are legal or ethical restrictions on sharing data publicly. For more information on unacceptable data access restrictions, please see <http://journals.plos.org/plosone/s/data-availability#loc-unacceptable-data-access-restrictions>. In your revised cover letter, please address the following prompts:

b) If there are no restrictions, please upload the minimal anonymized data set necessary to replicate your study findings as either Supporting Information files or to a stable, public repository and provide us with the relevant URLs, DOIs, or accession numbers. For a list of acceptable repositories, please see <http://journals.plos.org/plosone/s/data-availability#loc-recommended-repositories>. We will update your Data Availability statement on your behalf to reflect the information you provide.

2. Response: As instructed, we have uploaded a minimal anonymized data set necessary to facilitate replication of the study findings as a Supporting Information file.

3. Please amend your list of authors on the manuscript to ensure that each author is linked to an affiliation. Authors’ affiliations should reflect the institution where the work was done (if authors moved subsequently, you can also list the new affiliation stating “current affiliation:….” as necessary).

3. Response: We have checked this and provided only the affiliation where the work was done.

4. Ethics statement only appears at the end of the manuscript:
Your ethics statement should only appear in the Methods section of your manuscript. If your ethics statement is written in any section besides the Methods, please move it to the Methods section and delete it from any other section. Please ensure that your ethics statement is included in your manuscript, as the ethics statement entered into the online submission form will not be published alongside your manuscript.

4. Response: The ethics statement has been moved to the Methods section (p. 10)

**Reviewers' comments:**

Reviewer #1: "Successful outcomes with low–threshold intervention for cannabis use disorders in Norway - an observational study."

1. The labels “low-threshold” and “low-intensity” are used to describe the intervention. Are these appropriate given this is a 15-session, therapist-led intervention including a family session, and with an emphasis on achieving abstinence from cannabis use?

1. Response: We believe that “low-threshold” is an appropriate description, because no referral was needed, and participants could contact the service directly. We acknowledge that “low-intensity” may not be an adequate description; therefore, we have removed this term from the sentence on p. 20.

2. The intervention is described as “a secondary prevention intervention” – however the participants already perceived their cannabis use as problematic in this study (and had SDS scores ≥4). Should this still be described as a preventative intervention in this case?

2. Response: The term “secondary prevention” was used according to the following definition: “interventions that aim to reduce the impact of a disease or injury that has already occurred”.^[1]^ However, we did not consider this an important sentence; therefore, we have removed it to avoid confusion (p. 5).

Methods:
3. Phase 1 of the intervention is described as preparing for physical withdrawal, and as a “medical” phase. Did this include any type of pharmacological intervention? If so, details of these would be needed.

3. Response: No pharmacological intervention was utilized in any part of the study. The “medical phase” referred to a physical withdrawal. We have now inserted this definition in the description in the text (p. 6) and in Table 1 (p. 7).

Results:
4. The change in cannabis use from T0-T2 is unclear, in the main text: “the mean reduction was −12.6 days (95% CI = −16.4 to 8.8, p = 0.001)”. These confidence intervals would indicate a non-significant change. Is this an error?

4. Response: Thanks for this observation. We had omitted a minus sign in the confidence interval. This has now been corrected to: 95% CI = −16.4 to −8.8, p = 0.001 (p. 13).

5. I can’t see any data presented on how many participants were abstinent at T0. Given it is mentioned that: “They were advised to reduce or quit their cannabis use before starting the program, or during the first part of the program.”
How many were abstinent at the start of treatment? Why wasn’t baseline cannabis use adjusted for in the multivariable regression analysis?

5. Response: The number of participants that were abstinent at T0 is shown in Table 3 (N=5 participants). To examine changes in cannabis use, we used the pairwise t-test; therefore, the baseline data were integrated into the analysis. Instead of actual cannabis use, we used the perceived severity of cannabis use score (SDS) in the multivariable regression, because we considered this variable preferable for reflecting the severity of the substance use problem.

6. The authors describe conducting “an attrition analysis” – which analysis was conducted?

6. Response: The attrition analysis was based on all the variables listed in Table 2. This has now been clarified in the text (p. 12).

7. The results should follow the outline given on the clinical trial registration. ‘Other drug use’ is presented with the primary outcome in the manuscript which is not appropriate as this was not listed as a primary or secondary outcome on the registration. Data on alcohol/other drug use could be moved to a section labelled “exploratory analysis”.

7. Response: The results for “other drug use” have been moved to the section on secondary outcomes. We changed the heading to indicate that this section describes exploratory analyses (p. 14).

8. In Table 3 – what is the reason for writing “Ns” instead of the p-values that were >.05? These tables would also be improved by the addition of 95% confidence intervals. Table 3 needs to define what c refers to.

8. Response: As suggested, we have replaced the Ns with p-values in Table 3. We also note that we have corrected a minor error discovered in this table; the “other drugs” variable has now been reported in centimetre as it should be (it was reported in millimetres). We acknowledge that it would have been useful to insert the mean difference between the time-points with the related 95% CI, but we realized that this data would make the tables (Table 3 and 4) too massive: two more broad columns would be needed in each table. Therefore, we hope it is satisfactory to omit these columns. In table 3, we have omitted the “c”, because it was redundant at this point.

9. The text states “At the 3-month follow-up (T2), 59 respondents (58%) were contacted...”; If I am reading this correctly, why were nearly half of participants not contacted for this follow-up? Or does this number refer to the number of individuals that completed the follow-up?

9. Response: We attempted to contact all participants, but only 59 could be reached. We have revised the sentence to clarify this point (p. 12).

10. What are “classical cannabis products” used by participants? The background suggests that resin may be partly responsible for increased admissions to treatment, do you have data on herbal/resin use in this sample?

10. Response: We did not have data on herbal/resin use. The term “classical cannabis products” was meant to contrast with synthetic cannabis, as shown in Table 2. We have removed this phrase in the revised manuscript to avoid confusion (p.11).

11. When describing the sample characteristics, percentages or count would be better than “one in four”, “three of four” etc.

11. Response: We have reworded the findings to highlight the salient features (p. 11).

Discussion:

12. A brief sentence describing the intervention prior to describe the main findings about retention and cannabis use would improve readability of the discussion.

12. Response: We have inserted “CCP” to specify the intervention. This intervention was described in the Methods section (p. 16).

13. The reference provided to support not employing a waiting-list control (Sinadinovic, 2020) does not seem to noticeably support this suggestion. This point needs to be expanded, as this is a key criticism of the current investigation.

13. Response: Sinadinovic (2020) discussed this point in their limitations section:

“Determining the follow-up period is a balancing act between methodological design and the ethical issue of having participants randomized to a wait-list control group for extended periods, while having identified problematic cannabis use, but not being offered immediate help for this.”

Our study was completed before the Sinadinovic study, and our pre-study discussion about ethical issues came to a different conclusion. Thus, we chose an observational study. In future studies, a potential way to address this ethical dilemma might be to allocate half of the respondents to, for example, an internet-based CCP. A digital intervention became available after the present study was completed. We have elaborated more on this issue in the discussion (p. 20).

14. Further clarity and discussion are needed of the implications related to: “These professionals included only a proportion of those that were enrolled in the CCP in participating municipalities. We lacked information about the pre-study procedures; e.g., whether the professionals included all their eligible individuals in the study.”

14. Response: We apologize for the ambiguity in this statement: “These professionals included only a proportion of those that were enrolled in the CCP”. The word “those” referred to the fact that some eligible professionals did not participate in the study. Thus, we have no complete record of all the subjects that received CCP in the respective municipalities. The professionals that agreed to participate did so on a voluntary and idealistic basis. To the best of our knowledge, they followed the procedures for inclusion to the study, and invited all their own eligible participants. However, the study did not include a method for monitoring recruitment. Therefore, we did not have confirmatory data that we could include in a “flow-chart”, which indicated, for example, how many were excluded due to the exclusion criteria and how many were invited, but declined to participate. We have revised and simplified this paragraph to clarify the intended meaning (p. 20).

1. https://www.iwh.on.ca/what-researchers-mean-by/primary-secondary-and-tertiary-prevention
